# Specific Elimination of Latently HIV-1 Infected Cells Using HIV-1 Protease-Sensitive Toxin Nanocapsules

**DOI:** 10.1371/journal.pone.0151572

**Published:** 2016-04-06

**Authors:** Jing Wen, Ming Yan, Yang Liu, Jie Li, Yiming Xie, Yunfeng Lu, Masakazu Kamata, Irvin S. Y. Chen

**Affiliations:** 1 Department of Microbiology, Immunology, and Molecular Genetics, David Geffen School of Medicine at University of California, University of California Los Angeles, Los Angeles, California, United States of America; 2 Division of Hematology-Oncology, David Geffen School of Medicine at University of California Los Angeles, Los Angeles, California, United States of America; 3 Department of Biomolecular and Chemical Engineering, University of California Los Angeles, Los Angeles, California, United States of America; 4 California NanoSystems Institute (CNSI), University of California Los Angeles, Los Angeles, California, United States of America; 5 UCLA AIDS Institute, Los Angeles, California, United States of America; Jackson Laboratory, UNITED STATES

## Abstract

Anti-retroviral drugs suppress HIV-1 plasma viremia to undetectable levels; however, latent HIV-1 persists in reservoirs within HIV-1-infected patients. The silent provirus can be activated through the use of drugs, including protein kinase C activators and histone deacetylase inhibitors. This “shock” approach is then followed by “kill” of the producing cells either through direct HIV-1-induced cell death or natural immune mechanisms. However, these mechanisms are relatively slow and effectiveness is unclear. Here, we develop an approach to specifically target and kill cells that are activated early in the process of virus production. We utilize a novel nanocapsule technology whereby the ricin A chain is encapsulated in an inactive form within a polymer shell. Specificity for release of the ricin A toxin is conferred by peptide crosslinkers that are sensitive to cleavage by HIV-1 protease. By using well-established latent infection models, J-Lat and U1 cells, we demonstrate that only within an HIV-1-producing cell expressing functional HIV-1 protease will the nanocapsule release its ricin A cargo, shutting down viral and cellular protein synthesis, and ultimately leading to rapid death of the producer cell. Thus, we provide proof of principle for a novel technology to kill HIV-1-producing cells without effects on non-target cells.

## Introduction

Through the use of highly effective anti-retroviral drugs, acquired immune deficiency syndrome (AIDS) has become a manageable chronic disease for many patients [1,2]. However, latent HIV-1 reservoirs are still present in a small fraction of infected cells, memory T-cells and possibly other cell types [3,4,5]. These reservoirs sustain as silent integrated provirus [6], which can be activated through natural processes or through administration of drugs such as histone deacetylase (HDAC) inhibitors [7], protein kinase C (PKC) activators [8,9], positive transcription elongation factor b (p-TEFb) releasing agents [10,11] and second mitochondria-derived activator of caspase (Smac) mimetics [12]. Thus, a number of new drug regimens have been tested that are designed to induce latent HIV-1 reactivation, allowing recognition and clearance of the reactivated cells by the immune system [13]. This so-called “shock and kill” approach requires activators as well as effective means to eliminate those cells producing HIV-1 [14]. A large number of studies have been devoted to development of novel and effective activators, and some have been tested in clinical studies and have achieved an effect on HIV-1 reservoir reactivation [15,16]. In most of these studies, the clearance of cells producing activated HIV-1 has relied upon HIV-1 induced cell death or natural immune mechanisms, though these are relatively slow and insufficient processes [17]. Without an active means to kill cells producing HIV-1, infectious virus can be produced. In theory new virus spread upon reactivation is prevented from infection through the use of ongoing anti-retroviral therapy. However, there is evidence that HIV-1 can spread even under treatment with antiretroviral drugs, through cell-cell infection and in reservoir sites that are less penetrated by the drugs [18,19].

Several adjuvant strategies have been studied to improve the elimination of HIV-1 latent reservoirs after reactivation with activators. HIV-1 therapeutic vaccines have gained renewed interest in either accelerating the decay of the activated cells during ART or improving the control of viral rebound after ART interruption [20,21]. Several HIV-1 therapeutic vaccines have been tested in clinical trials; however, none of them have prolonged viral suppression in infected individuals after ART interruption [20]. Passive immunotherapy with broadly neutralizing HIV-1-specific antibodies is also being considered [22,23,24]. One phase I study of passive immunization with neutralizing antibodies directed at CD4 binding sites showed that the treatment transiently reduces HIV-1 viral loads in humans [25]. However, this antibody administration required an intravenous dosage as high as 30 mg/kg. Moreover, potential obstacles include the limited accessibility of broadly neutralizing antibodies to certain anatomic reservoir sites, immunogenicity, and emergence of viral escape mutants [6,26]. Inhibitors of the interaction between PD-1 and its ligands have shown efficacy in cancer treatment, so the blockade of immune checkpoint molecules are also being explored as a potential strategy [27,28]. Thus, alternative means to rapidly eliminate the activated cells prior to release of virus is desirable.

We adapted a technology whereby individual protein molecules are encapsulated within a thin polymer shell, termed nanocapsules [29]. These nanocapsules can effectively enter the cells, owing to the positive charge on their surface and release their protein cargo due to the “proton-sponge” effect [30,31] and cation-mediated membrane destabilization from the postively charged monomer. One unique advantage of this nanocapsule platform is its flexibility. By altering the chemical properties of the nanocapsule surface, one can modulate critical factors such as cell surface affinity, immunogenicity, release rates of its cargo, circulation time and biodistribution. By incorporating responsive components into stabilizing crosslinkers of the polymer shell, the cargo can be released controllably in response to cellular environments such as endosomal low pH [32]or cellular proteases [33,34]. Here, we modified the crosslinkers to develop a new class of nanocapsules containing toxins engineered to specifically release their cargo within cells latently infected with HIV-1 upon reactivation and rapidly kill those cells.

## Materials and methods

### Ethics Statement

All studies described in this manuscript have approval by the UCLA Institutional Review Board (IRB).

### Nanocapsule synthesis

Centers for Disease Control and Prevention (CDC) and UCLA Institutiaonal Biosafety Committee (IBC) do not consider ricin A chain as a selective toxin. Ricin A (100 μg) was dissolved into 250 μL of 10 mM pH 8.5 sodium bicarbonate buffer to form a protein solution. First, the positively charged monomer *N*-(3-aminopropyl) methacrylamide (APmTAAm), prepared in a 10 mg/mL aqueous solution, was added to the protein solution while stirring. Then the neutral acrylamide (AAm) monomer and bisacryloylated crosslinkers were added sequentially to the protein solution while stirring. The molar ratio of AAm:APM:crosslinker was adjusted to 5:5:1. Radical polymerization was initiated by adding both ammonium persulfate (1:10 molar ratio of total monomers) dissolved in deionized water and the same volume of 10% *N*,*N*,*N'*,*N'*-tetramethylethylenediamine into the reaction solution. The polymerization was allowed to proceed for 90 min in a nitrogen atmosphere at 4°C. Finally, unreacted monomers, crosslinkers, and initiators were removed by dialysis in 10 mM pH 7.4 phosphate buffer. A ten times greater amount of monomer was required when the reaction volume was less than 50 μL and the final protein concentration was lower than 0.05 μg/μL.

### Peptide crosslinker modification

The sequences of the peptide are shown in S1 Table. A peptide crosslinker was obtained by reaction between the amine groups of the peptide and *N*-acryloxysuccinimide at a 1:2 molar ratio at pH 8 for 2 hours at room temperature. The resulting solution was dialyzed overnight against H_2_O to remove unreacted substrates, lyophilized overnight, and then stored at -20°C.

### Cells

Latently infected T-lymphoid J-Lat 10.6 clone (Catalog Number 9849) [35] and monocytic U1 cells (Catalog Number 165) [36] were provided by the NIH AIDS Reagent Program. Jurkat cells were purchased from American Type Culture Collection (ATCC) and transduced a lentiviral vector stably expressing blue fluorescent protein (BFP) to obtain control Jurkat cells expressing BFP. The 293-Affinofile cell line was generously provided by Dr. Benhur Lee. 293-Affinofile cells were transduced with an HIV-1 envelope-pseudotyped luciferase reporter virus, which was generated with the luciferase-encoding HIV-1 vector pNL-Luc.AM and envelope expression vector BaL.26, to obtain HIV-1-infected 293-Affinofile cells [32].

### Cell death monitored by flow cytometry

Cell numbers were measured on MACSQuant flow cytometer. Same volumes of cell culture were taken and fixed in 2% formaldehyde. Cell numbers were counted by flow cytometer in exactly the same volume. Specific killing induced by nanocapsules in different cell populations was monitored on BD Fortessa flow cytometer. After 4 hours of transduction with samples, the cells were reactivated with 10 μM prostratin. Mixed Jurkat cells, having J-Lat and control Jurkat cells, were transduced with nanocapsules, and EGFP and BFP intensities were monitored by flow cytometry on Day 1 and Day 2 after prostratin reactivation. U1 cells were stained with 1:1500 diluted anti-p24 antibody (KC57-RD1) for 1 hour at room temperature, and monitored by flow cytometry. HIV-1 PR inhibitor Indinavir Sulfate (IDV) was added for 18 hours pretreatment before reactivation and applied during the reactivation with a concentration of 10 μM.

## Results

### Nanocapsules bearing ricin A with nanometer size and positive surface charge

**Fig 1:**
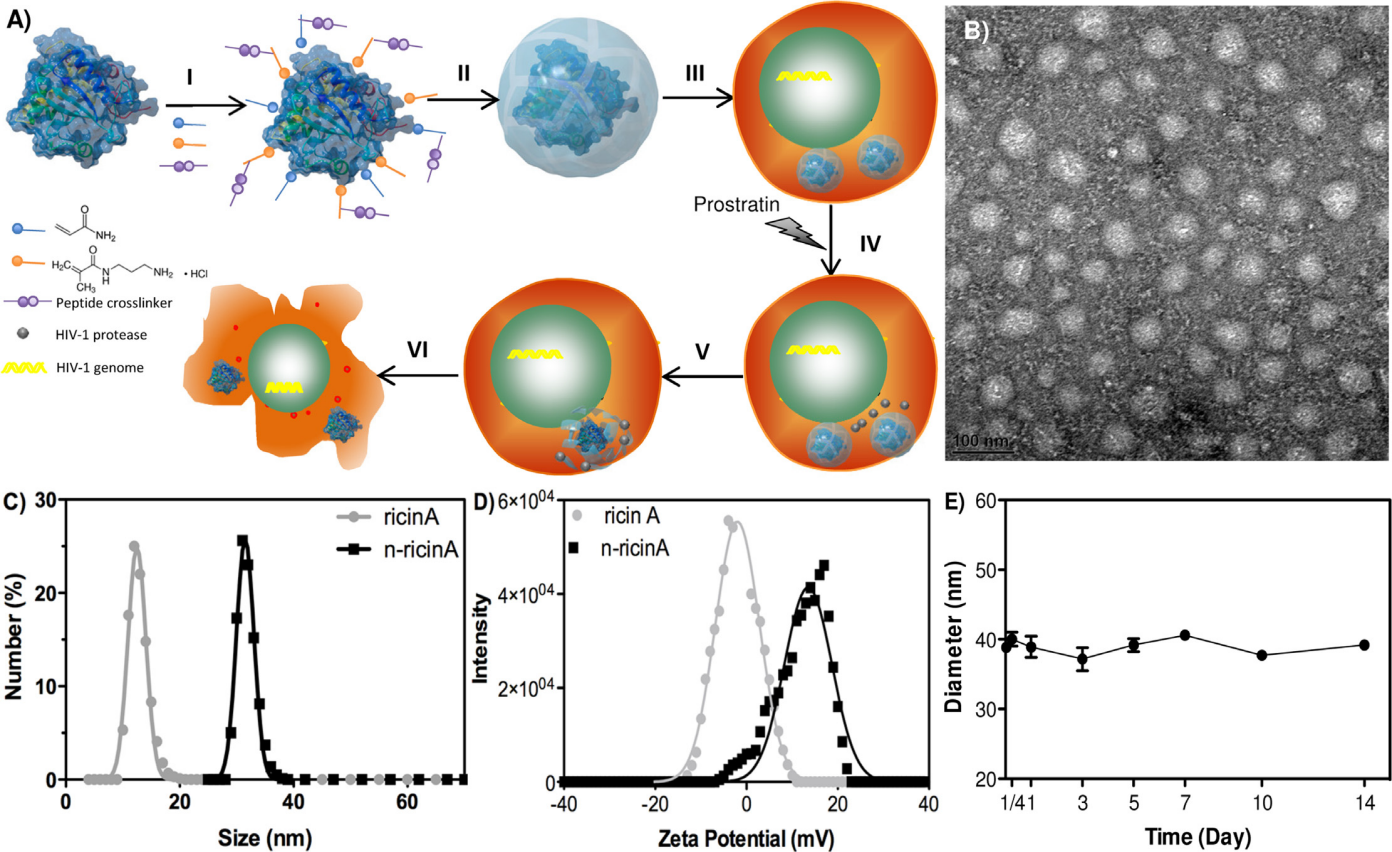
The formation of ricin A nanocapsules (n-ricinA). A) Illustration of the synthesis, delivery and function of HIV-1 PR-sensitive nanocapsules: I) self-assembly of positive monomer A, hydrophilic monomer B and HIV-1 PR-sensitive crosslinker C on the surface of ricin A molecules; II) formation of HIV-1 PR-sensitive n-ricinA; III) delivery; IV) reactivation by drugs; V) specific release upon intracellular HIV-1 PR and VI) killing only the reactivated cells. B) TEM image of n-ricinA (Scale bar = 100 nm). C) Particle size distributions and D) surface charge (zeta potential) of native ricinA and n-ricinA. E) Stability of n-ricinA in PBS at 4°C for two weeks. Size of n-ricinA was monitored by Dynamic light scattering.

Ricin A is the A chain of the ricin toxin, which specifically and irreversibly hydrolyses the N-glycosidic bond of the adenine residue at position 4324 within the rRNA, which is important in binding elongation factors during protein synthesis [37,38]. The depurination event by ricin A rapidly and irreversibly inactivates ribosomal activity, resulting in toxicity from inhibition of protein synthesis [39]. For cytotoxic function, ricin A must be delivered into the cytoplasm by the ricin B chain. The ricin A chain by itself is incapable of entering cells and has been shown to be non-toxic in animal models [40,41,42,43]. We engineered a ricin A nanocapsule delivery system designed to release its cargo only within HIV-1 activated cells. This is achieved by encapsulating each ricin A molecule within a polymer shell to form nanocapsules, whose peptide crosslinkers were designed to be cleavable by HIV-1 protease (HIV-PR) [44], an aspartic protease responsible for cleavage of viral gag-pol precursor protein [45]. While HIV-1 PR is typically present in maturing virions, several studies report active HIV-1 PR within the infected cells [46,47,48]. The general concept for the nanocapsule platform is as follows. As illustrated in Fig 1A, positive monomer A, hydrophilic monomer B and crosslinker C were enriched on the surface of ricin A molecules through electrostatic interaction and hydrogen bonding (step I); subsequent *in situ* polymerization leads to the growth of a thin layer of polymer shell around the ricin A molecules and formation of ricin A nanocapsules (denoted as n-ricinA) (step II). n-ricinA are efficiently internalized by cells due to their positive charge and remain stable inside the cells following endocytosis (step III). Upon the reactivation of latently infected HIV-1 reservoirs (step IV), HIV-1 PR are produced (step V) and activates the n-ricinA by cleaving the crosslinkers leading to release of the ricin A cargo (step VI), which then leads to rapid death of HIV-1 infected cells (step VII). To determine the morphology of nanocapsules, n-ricinA was characterized by transmission electron microscropy (TEM), revealing a round morphology with a uniform size of 45±5 nm (Fig 1B). Consistent with the TEM image, the hydrodynamic size of n-ricinA confirmed by dynamic light scattering (DLS) is (37.9±8.2) nm, an obvious increase in size compared to native ricin A molecules of (13.3±1.5) nm (Fig 1C). The polymer shell also changes the surface charge of ricin A from negative (~-5 mV) into positive (~+15 mV), which facilitates its internalization by cells (Fig 1D). Furthermore, at physiological conditions in phosphate-buffered saline (PBS) buffer, n-ricinA formulated with HIV-1 PR sensitive peptide crosslinkers were stable in PBS solution at 4°C without aggregation or degradation in two weeks (Fig 1E).

### Minimal non-specific cytotoxicity from nanocapsules containing ricin A

**Fig 2:**
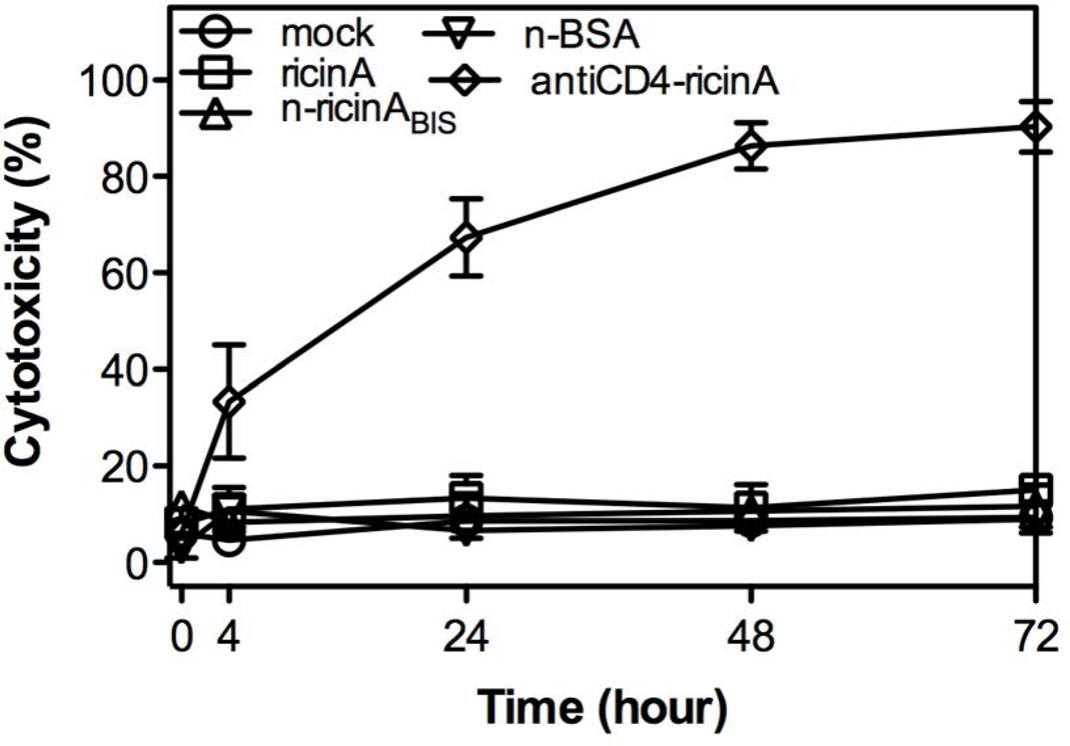
Minimal non-specific cytotoxicity of n-ricinA. Dead cells of J-Lat were removed by MACS^®^ LS columns. Dead-cell-removed J-Lat cells were transduced with 1 μM of native ricinA, n-ricinA_BIS_ (ricin A nanocapsule with non-degradable crosslinker), n-BSA (BSA (bovine serum albumin) nanocapsule with HIV-1 PR cleavable peptide as crosslinker), and antiCD4-ricinA (immunotoxin anti-CD4 conjugated ricinA), respectively. Cell cytotoxicity was determined with CytoTox-Glo^TM^ cytotoxicity kit (Promega) using a plate reader. Cytotoxicity was calculated by dividing the dead cell luminescence by the total cell luminescence. Error bars: Mean±SD.

Non-specific toxicity is the major obstacle to applying ricin for HIV-1 therapy [49]. Without the HIV-1 PR trigger, n-ricinA are shielded by the polymer shell to minimize non-specific toxicity. Non-specific toxicity was tested in J-Lat, a latently HIV-1-infected Jurkat cell line, without reactivation [35]. To further control for the effects of the polymer shell harboring unreleased ricin A, ricin A nanocapsules were synthesized with a non-degradable crosslinker (BIS) denoted ‘n-ricinA_BIS_’. As a control protein, a nanocapsule encapsulating bovine serum albumin (BSA) denoted as ‘n-BSA’ was also prepared. Immunotoxin antiCD4-ricinA, which showed efficient killing of CD4+ cells, was prepared by linking monoclonal anti-CD4 antibody to ricin A through a thioether linkage [50], which served as a positive control. Levels of cytotoxicity were evaluated by Cyto-tox Glo assay at serial time points after 4h transduction with native ricin A (ricinA), n-ricinA_BIS_, n-BSA or antiCD4-ricinA (Fig 2). Note that both ‘n-ricinA_BIS_’ and n-BSA showed similar cell cytotoxicity to ricinA, which is deficient for entering cells but retains catalytic activity. On the other hand, antiCD4-ricinA induced cell death within 4 hours. These results show that the polymer shell of the nanocapsules did not exhibit detectable cytotoxicity and shielded the activity of ricin A.

### Specific release by HIV-1 PR cleavage *in vitro*

**Fig 3:**
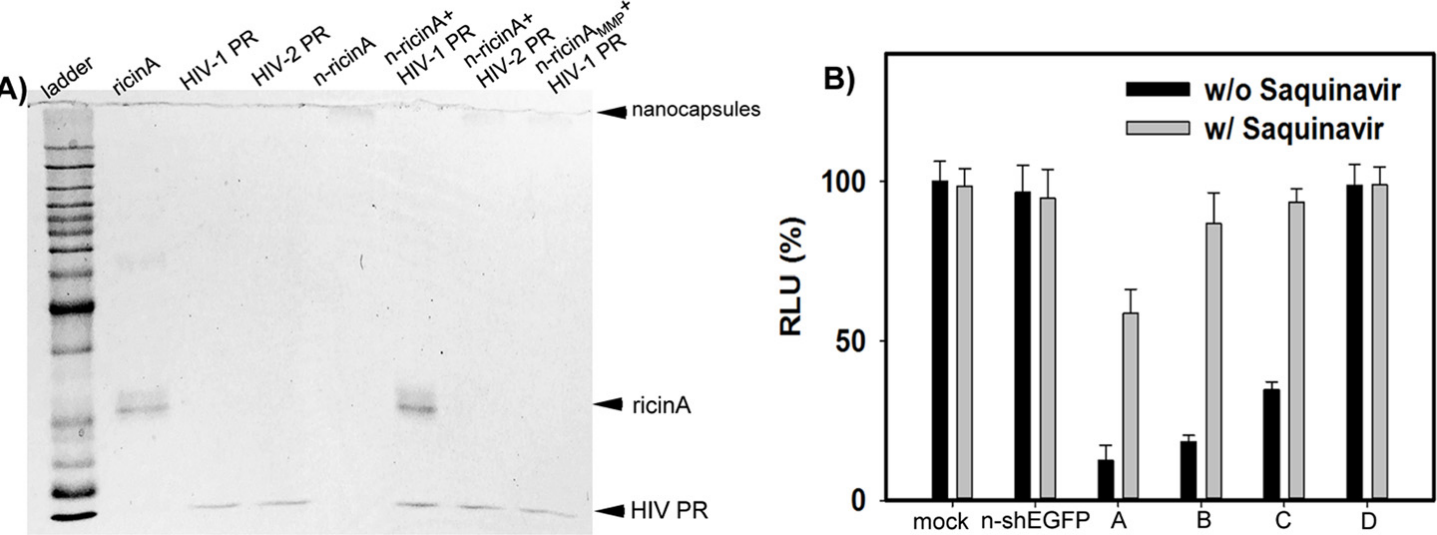
Specific release of n-ricinA to HIV-1 PR protein treatment *in vitro*. A) SDS-PAGE of ricin A released from nanocapsules with protease treatment. Samples were treated with or without 50 nM protease (HIV-1 PR or HIV-2 PR) in protease working condition at 37°C for 2 hours, and applied to SDS-PAGE, followed by Coomassie Brilliant Blue staining. B) Knockdown of luciferase in HIV-1-infected 293-Affinofile cell by shLuc DNA cassette released from HIV-1-PR sensitive DNA nanocapsules with different ratios (A-3:0; B-2:1; C-1:2 and D-0:3) of HIV-1-PR-senstive to non-degradable crosslinker. Error bars: Mean±SD.

To demonstrate the specificity of release in response to HIV-1 PR, we first tested the n-ricinA susceptibility to recombinant HIV-1 PR. As a control, we constructed a nanocapsule where the crosslinker could not be cleaved by HIV-1 PR but could be cleavable by another protease, matrix metalloproteinase (MMP) (denoted as n-ricinA_MMP_). Fig 3A shows a SDS-PAGE image of ricin A released from n-ricinA after treatment by HIV-1 PR or HIV-2 PR, using as a negative control. Ricin A is only released after treatment with HIV-1 PR (n-ricinA + HIV-1 PR), but not with HIV-2 PR (n-ricinA + HIV-2 PR), which has a different peptide substrate specificity. HIV-1 PR treatment of the n-ricinA_MMP_ could not release the ricin A cargo (n-ricinA_MMP_ + HIV-1 PR), further demonstrating the specificity of release requiring HIV-1 PR and its peptide substrate.

We next confirmed that HIV-1 PR-sensitive nanocapsules were able to release the cargo in HIV-1 infected cells. In order to demonstrate that the nanocapsule cargo is released in HIV-1 infected cells as a result of HIV-1 PR expression, HIV-1 PR-sensitive nanocapsules were constructed, not with ricin A, but with a DNA cassette encoding an shRNA directed to luciferase [32]. Affinofile cells were infected with HIV-1 bearing a luciferase reporter gene and the cells were then transduced with nanocapsules bearing the DNA cassette (n-shLuc) [51]. Only if the DNA cassette is released and shRNA transcribed within the cell will luciferase activity from HIV-1 be ablated. Thus, this serves as a sensitive means to assess the activity and specificity for targeting of n-shLuc to HIV-1 infected cells. In order to control the release rate, we constructed nanocapsules using four different ratios of HIV-1 PR-cleavable to non-degradable crosslinker (A-3:0; B-2:1; C-1:2 and D-0:3) (Fig 3B). The nanocapsule synthesized with only HIV-1 PR cleavable crosslinker (A-3:0) resulted in the greatest reduction of luciferase activity, indicating specific and timed release of the DNA cassette and cleavage of the luciferase reporter encoded within HIV-1. This reduction of luciferase activity was substantially attenuated by adding a protease inhibitor, Saquinavir, indicating that the luciferase ablation was a result of HIV-1 PR activity within the infected cells. Decreasing ratios of HIV-1 PR cleavable crosslinker (B, C, and D) resulted in correspondingly reduced levels of luciferase knockdown. With no HIV-1 PR cleavable crosslinker (D-0:3), we observed no knockdown of luciferase activity; insufficient DNA cassette is released to knockdown the HIV-1 luciferase reporter. In all cases where luciferase activity was ablated, the knockdown was attenuated by addition of the HIV-1 PR inhibitor. Thus, these results demonstrate that the HIV-1 PR expressed following HIV-1 infection of cells is sufficient to release the nanocapsule cargo, which in this case, was an shRNA directed to luciferase to knockdown HIV-1 gene expression.

### Rapid cell death of reactivated HIV-1 infected cells induced by n-ricinA

**Fig 4:**
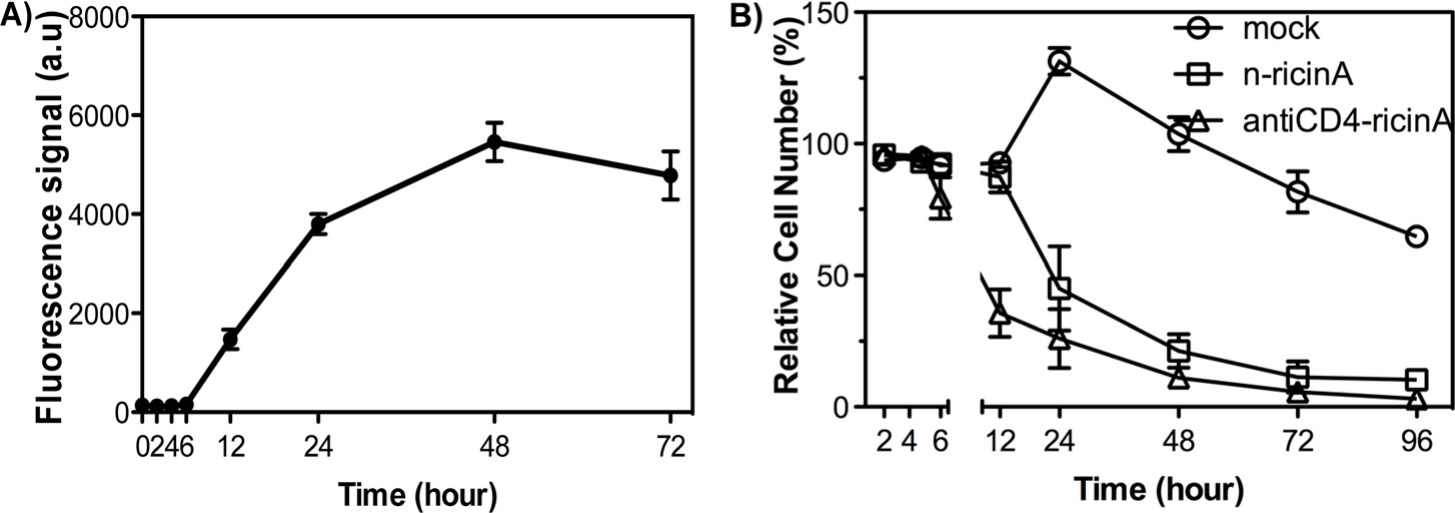
Rapid killing of reactivated HIV-1 infected cells by n-ricinA. A) A time course of HIV-1 PR expression in J-Lat after prostratin reactivation. A FRET peptide substrate of HIV-1 PR was added to cell lysate to monitor HIV-1 PR activity with the fluorescence signal at 490 nm. B) A time course of the relative cell numbers of J-Lat after prostratin reactivation without transduction, with transduction of n-ricinA, or with incubation of antiCD4-ricinA. Error bars: Mean±SD.

Based upon the above results, we predict that a similarly formulated nanocapsule bearing HIV-1 PR-cleavable crosslinker and carrying ricin A would specifically kill cells expressing HIV-1 PR. Having confirmed that the nanocapsules can release the cargo in the presence of HIV-1 PR, we used the J-Lat cell model system to assess the efficacy of ricin A killing in cells induced to express HIV-1. We first confirmed intracellular HIV-1 PR activity in reactivated J-Lat cells with a fluorescence resonance energy transfer (FRET)-peptide substrate specific for HIV-1 PR, at serial time points after reactivation of provirus by prostratin. fluorescence intensity from the substrate increased; active HIV-1 PR began to be detected in J-Lat cells at 12h after reactivation (Fig 4A). In parallel with this, the HIV-1 p24 antigen, as a measure of HIV-1 produced, could be observed in culture supernatants from 12h (S1 Fig). We further tested the kinetics of cell death induced by n-ricinA (100% HIV-1 PR-cleavable crosslinker equivalent to 3:0 ratio of Fig 3B) based on cell numbers after reactivation (Fig 4B). The number of cells treated with antiCD4-ricinA decreased starting as early as 6h; moreover, in 2 days, the cell viability decreased to less than 5%. Based on previous studies [52,53], the antiCD4-ricinA immunotoxin rapidly inhibits protein synthesis and begins to kill the cells. In contrast, n-ricinA started to kill cells after 12h, in accord with requirement of HIV-1 PR activity to release the ricin A. The mock cells without transduction died due to cytotoxicity from HIV-1 reactivation, but required more than 4 days to reach 50% cell death.

### Specific killing of reactivated latently HIV-1 infected cells expressing HIV-1 by n-ricinA

**Fig 5:**
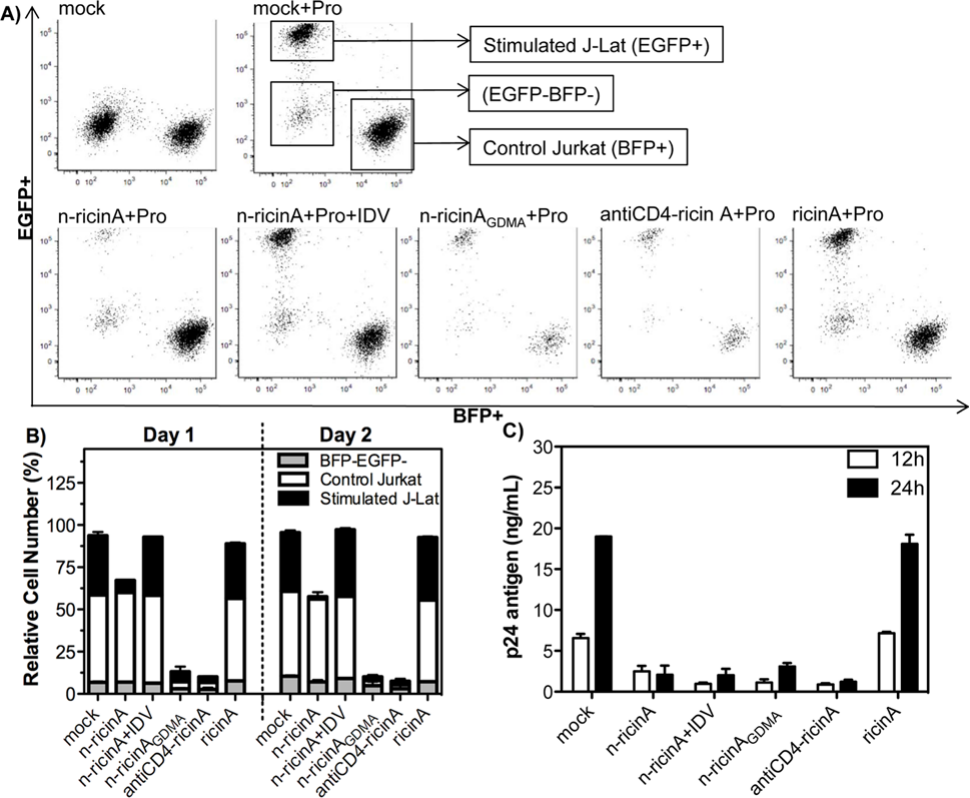
Specific elimination of J-Lat cells by n-ricinA after reactivation. A) Specific elimination of J-Lat cells with reactivation of provirus. J-Lat cells and control Jurkat expressing BFP were co-cultured at a 1:1 ratio. Cells were transduced with n-ricin, n-ricinA_GDMA_, antiCD4-ricinA, or ricinA for 4 hours. Cells were then treated with 10 μM prostratin (Pro) for reactivation of provirus. With reactivation, three populations, reactivated J-Lat (EGFP+), control Jurkat (BFP+), and unreactivated Jurkat cells (EGFP-BFP-), were clearly separated by fluorescence. The cells with Indinavir sulfate (IDV) treatment were pretreated with 10 μM IDV for 18 hours before transduction. B) Relative cell numbers of these three populations in J-Lat cells were further analyzed both on Day 1 and Day 2 after reactivation. C) The production of virus from transduced J-Lat cells one day after reactivation. One million cells were cultured in 500 μL medium with 10 μM of prostratin, and p24 in the culture supernatant were quantitated by ELISA at 12 hours and 24 hours after reactivation. Error bars: Mean±SD.

Given the killing of HIV-1 infected cells without non-specific toxicity observed above, we further confirmed the specificity for killing of HIV-1 producing cells in a mixed population of HIV-1 infected and non-infected cells. We used the experimental system of J-Lat cells, in which HIV-1 expressing cells can be distinguished after reactivation from non-expressing cells by EGFP expression. A control Jurkat cell (expressing blue fluorescent protein, BFP) was mixed in equal proportion, as a control to assess specific killing. Besides antiCD4-ricinA, we constructed another positive-control nanocapsule, in which the crosslinker is cleavable at pH of less than 5.5 and the cargo will be released in all cells by endosomal low pH irrespective of HIV-1 PR expression (denoted as n-ricinA_GDMA_). Thus, n-ricinA_GDMA_ should induce cell death in all transduced cells. J-Lat and control Jurkat were mixed at a 1:1 ratio and transduced with n-ricinA, n-ricinA_GDMA_, antiCD4-ricin or ricinA. A similar transduction efficiency of n-ricinA in both J-Lat and control Jurkat was demonstrated by Rhodamine B-labeled n-ricinA (S2 Fig). Three populations were separated by fluorescence: reactivated J-Lat (EGFP+), control Jurkat (BFP+) and Jurkat in which content HIV-1 was not reactivated (EGFP-BFP-). As shown in Fig 5A, as expected, both n-ricinA_GDMA_ and antiCD4-ricinA showed non-specific cytotoxicity in all three-cell populations. In contrast, n-ricinA resulted in a reduction of the EGFP+ population only, indicating specific release of ricin A and killing only in the J-Lat cells with provirus reactivation. To quantitatively compare the killing preference, relative cell numbers of each population were compared (Fig 5B). Over 75% of reactivated J-Lat cells were killed within one day, while both inactive Jurkat and control Jurkat maintained similar percentages as that of mock. Similar reduction was also seen on Day2. To demonstrate that the killing is due to HIV-1 PR mediated release, a protease inhibitor, Indinavir (IDV), was added. The reduction of the EGFP+ population was nearly completely inhibited indicating that the killing was a result of HIV-1 PR activity within the cells having provirus reactivation (Fig 5A and 5B). The native ricinA did not show cytotoxicity in all cell populations similar to mock (Fig 5A and 5B).

In parallel HIV-1 p24 antigen in culture supernatants was measured at 12h and 24h after reactivation (Fig 5C). Both n-ricinA_GDMA_ and antiCD4-ricinA induced significant non-specific cytotoxicity including in reactivated J-Lat cells, resulting in a similar and low p24 level in the culture supernatants. With the transduction of n-ricinA, p24 antigen levels were 3-fold lower than that of mock at 12h after reactivation; in contrast, the relative cell number of this n-ricinA treated sample was not decreased until 24h (Fig 4B). These results demonstrated that n-ricinA inhibited HIV-1 protein synthesis within 12 hours and prior to cell death, consistent with the known mechanism of action for ricin A. Native ricin A protein treatment did not affect p24 levels, similar to that seen on mock.

**Fig 6:**
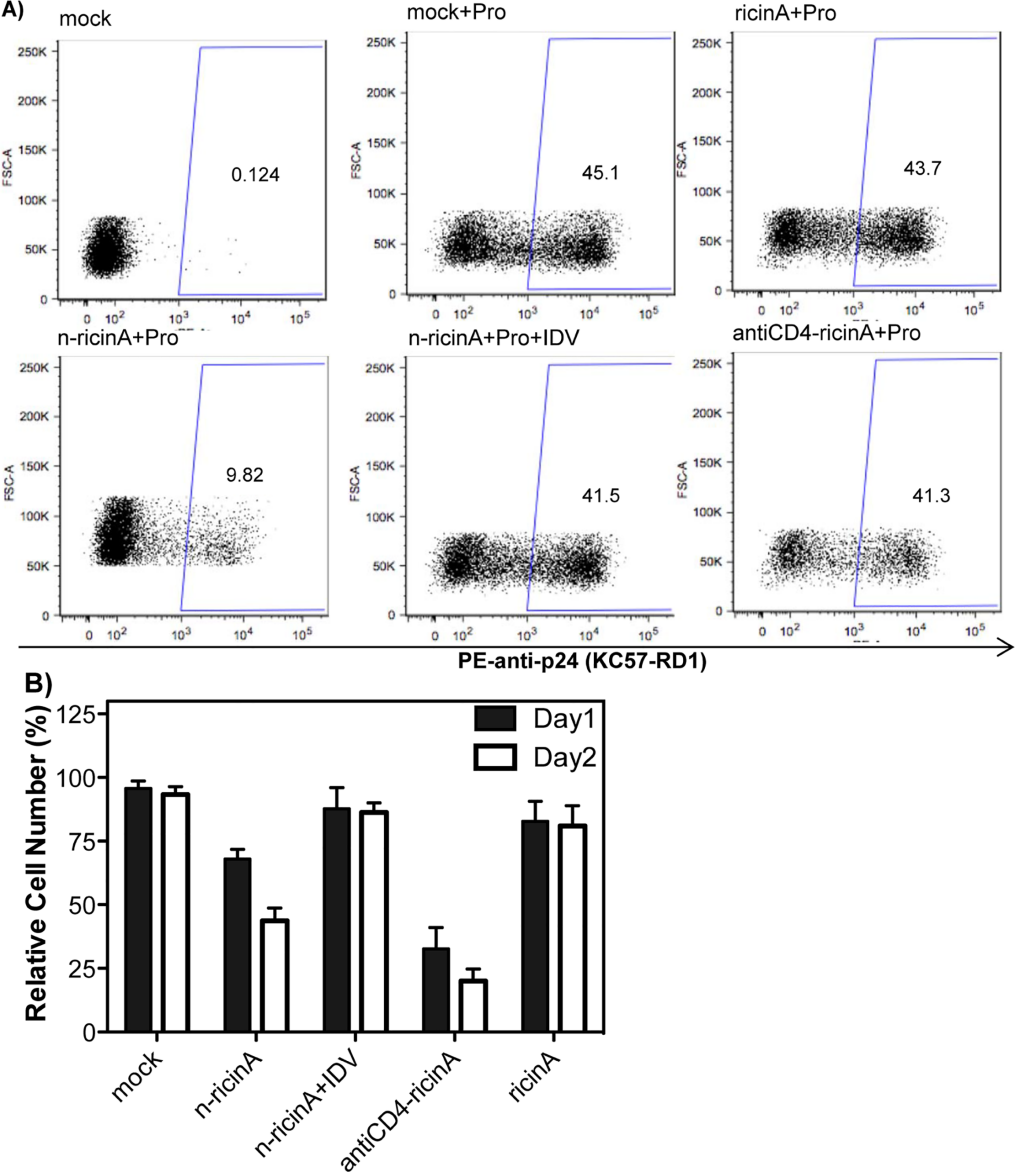
Specific elimination of U1 cells by n-ricinA after reactivation. A) Two days after 10 μM prostratin reactivation, levels of intracellular p24 antigen were analyzed by flow cytometry. B) Relative cell numbers of U1 cells transduced with ricinA, n-ricinA and antiCD4-ricinA on Day 1 and Day 2 after prostratin reactivation. Error bars: Mean±SD.

The specific killing of reactivated HIV-1 infected cells was also examined in the HIV-1 latently-infected monocytic U1 cell line [36]. The reactivation of latent HIV-1 was monitored by the level of intracellular p24-antigen. After treatment with prostratin, 25% of cells expressed intracellular p24 on Day1 (S3 Fig); on Day2 more than 45% of cells were positive for p24 gag (Fig 6A). As shown in Fig 6A, with the transduction of antiCD4-ricinA together with prostratin, the percentage of p24 positive cells was similar to that of mock, although total cell numbers were decreased (see Fig 6B). Compared to mock, n-ricinA showed an 80% decrease of p24 positive cells (9%). With the HIV-1 PR inhibitor IDV, the percentage of p24 positive cells transduced n-ricinA was comparable with that of mock, further demonstrating that HIV-1 PR triggered the decrease of p24 positive population by n-ricinA. As a negative control, ricinA did not change p24 levels after reactivation.

Relative cell numbers of U1 cells were measured on both Day1 and Day2 after reactivation (Fig 6B). Compared to mock, antiCD4-ricinA induced significant cell death on Day1. With the transduction of n-ricinA, 60% of reactivated cells were killed on Day1, which increased to 80% on Day2. Moreover, the decrease in cell numbers induced by n-ricinA was inhibited by the HIV-1 PR inhibitor. Moreover, the p24 antigen level of the cell supernatant transduced with n-ricinA was only one fifth of that of the mock cells (S2 Table). Our results demonstrated that n-ricinA induces specific cell death in p24 positive cells.

**Fig 7:**
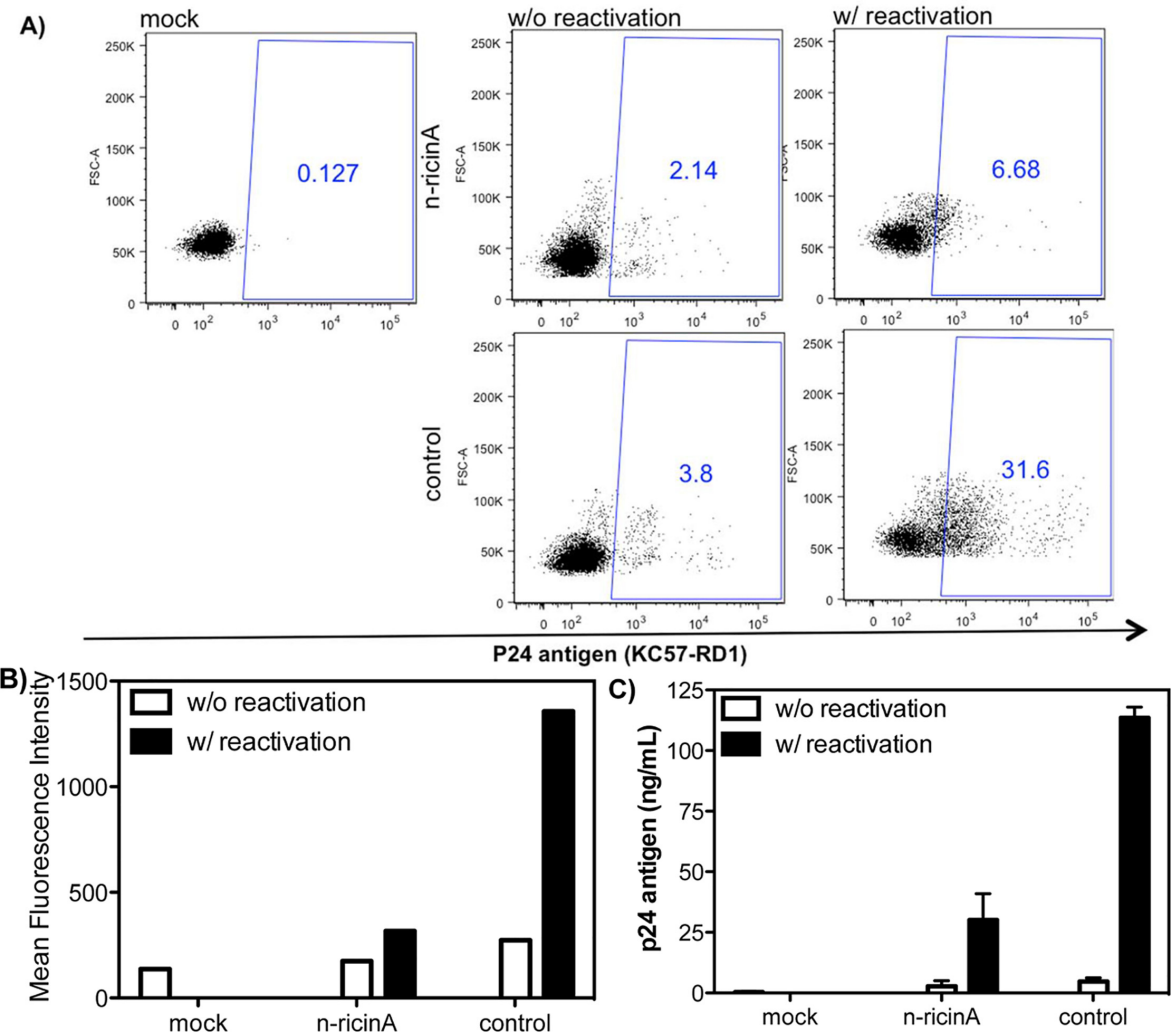
Elimination of latently infected primary T cells by n-ricinA after reactivation. A) 2x10^6^/mL of naïve CD4+ T cells were infected with 2000 ng/mL of wild type HIV-1 NL4-3 and cultured with 5 ng/mL of IL-7 and 5 ng/mL of TGFβ-1 for 2 weeks to obtain the latently infected primary T cells. Cells were then transduced with nanocapsule for 4 hours with or without 1μg/mL of CD3 and CD28 antibody reactivation for three days, levels of intracellular p24 antigen in latently infected primary T cells were stained by KC57-RD1 and analyzed by flow cytometry. B) Intracellular p24 levels of latently infected primary T cells with or without reactivation were measured based on flow cytometry results. Data are presented as the mean fluorescence intensity of p24 staining. C) The production of virus p24 from reactivated primary T cells in culture supernatant three days after reactivation. Mock: cells without virus infection. Control: infected cells not transduced with n-ricinA. Error bars: Mean±SD.

Similar results were seen using a primary cell reactivation system using primary T-cells and replication-competent HIV-1 (Fig 7A). Latently infected T-cells were prepared by infection of naïve CD4+ T-cells with HIV-1 NL4-3 followed by culture with IL-7/TGFβ-1 for 2 weeks, a condition modified based upon previous publications [54,55,56]. The cells were then transduced with n-ricinA and reactivated with CD3 and CD28. A significant reduction of p24 positive cells (Fig 7B) and p24 in supernatant (Fig 7C) was observed in n-ricinA transduced cultures.

## Discussion

Nanocapsules encapsulating ricin A with HIV-1 PR cleavable crosslinkers were evaluated for specific release in the presence of HIV-1 PR. We demonstrate that the nanocapsule cargo is released specifically in HIV-1 producing cells. Cells which harbor the HIV-1 virus in the latent form do not induce the release of the ricin A cargo, whereas after reactivation of HIV-1 in the same cells using prostratin, nanocapsules released ricin A to induce cell death in HIV-1 infected cells upon reactivation. Since ricin A rapidly shuts down protein synthesis, cells die more quickly than cell death induced by HIV-1. Importantly, native ricin A as well as ricin A nanocapsules do not show evidence of toxicity on non-infected cells. These studies provide proof of principle for the use of HIV-1 PR sensitive nanocapsules to deliver drugs that eliminate cells in which HIV-1 is reactivated from latency.

The persistence of latent HIV-1 in AIDS patients under highly active anti-retroviral therapy (HAART) is the primary reason why patients must remain on HAART and poses the major obstacle to an HIV-1 cure. “Shock and kill” is being considered as a promising strategy to purge HIV-1 reservoirs. Several agents have been investigated and shown to induce latent HIV-1 from infected lymphocytes. However, “kill” has relied upon HIV-1 induced cell death and/or natural host immune mechanisms. These means are likely to be either inefficient or insufficiently rapid before the virus can spread to neighboring cells [57]. Our data show that 50% of HIV-1 infected cell death occurs in 4 days, compared to 50% are killed by n-ricinA within 24 hours (Fig 4B). Furthermore, n-ricinA rapidly shuts off viral production, beginning at less than 12 hours (Fig 5C). Therefore, n-ricinA will rapidly limit virus production to prevent spread to uninfected cells. Even in the presence of anti-retroviral drugs, it has been proposed that HIV-1 can spread within tissue compartments that are poorly accessible to anti-retroviral drugs [58]. In addition, HIV-1 appears to spread more readily through cell-to-cell transmission in the presence of concentrations of a drug that effectively neutralizes virus to cell transmission [19]. Therefore, there is still an urgent need for new approaches that will rapidly kill those cells where HIV-1 is activated.

We previously developed a nanocapsule delivery platform for macromolecules that is more robust and effective than other methods [29,59]. We have adapted the technology for a number of anti-HIV-1 applications, including the delivery of RNAi to knockdown CCR5 and delivery of CRISPR/Cas9 gRNA delivery to ablate the HIV-1 provirus [32,60]. Here, we deliver a protein toxin to kill cells producing HIV-1. Briefly, protein is mixed with monomer and crosslinker molecules in buffer solution, and hydrogen bonding and electrostatic interactions enrich the monomer and the crosslinker around the protein molecules. Subsequent polymerization wraps the protein molecule in a thin polymer layer, leading to the formation of individual protein nanocapsules that consist of a protein core and a thin polymer shell. The crosslinkers can be pH sensitive, which are stable at physiological conditions (pH~7.4) but are degradable at pH<6; or enzyme sensitive, which only degrades under specific enzyme treatment. The polymer shell of nanocapsules shields its cargo from its non-specific side effect and the immune system, minimizing immunogenicity. In addition, due to relative ease of chemical manipulation, nanocapsules can be engineered to optimize delivery for relevant cell types by tuning the components of monomers for polymerization or introducing cell surface binding ligands. Here, we extended this platform to specifically release cargos in HIV-1 producing cells. We designed a novel nanocapsule encapsulating ricin A with HIV-1 PR-cleavable crosslinker, which is highly specific to HIV-1 producing cells with minimal effects on non-target cells. In the viral life cycle, HIV-1 PR is typically activated during the budding process where immature virions are converted to mature virions [45,61]. However, several studies report active HIV-1 PR within the infected cells [46,47,48]. We took advantage of this intracellular HIV-1 PR activity by designing peptide crosslinkers that are substrates for HIV-1 PR cleavage, and exploited the intracellular activity of HIV-1 PR to activate the nanocapsules and release its cargo. We demonstrate using ricin A as cargo that only those latently infected HIV-1 cells activated to produce HIV-1 are killed.

Immunotoxins are bi-functional proteins that combine a protein toxin with an antibody or a ligand [52]. Targeted killing of diseased cells by immunotoxins has been intensively studied for cancer [62]. While several clinical studies with Diphtheria toxin (DT), Pseudomonas exotoxin A (PE) and ricin A chain have shown potential, the clinical use of such immunotoxins has been limited by poor specificity, weak internalization, and/or immunogenicity [63,64]. Similarly, antibody-toxin conjugates have been proposed for AIDS. A CD4-PE molecule was have been intensively studied and used to treat AIDS in clinical trials in the 1990s without efficacy [49,65,66,67]. There is now renewed interest in the use of immunotoxins, targeted to HIV-1 infected cells as a means to purge reservoirs. Immunotoxins were constructed to direct to HIV-1 envelope expressed on the cell surface [68]. Studies revisiting PE fusions targeting gp120, CD4-PE and 3B3-PE (an affinity-matured Fab b12), show efficacy in humanized mouse models in combination with antiretroviral therapy (ART) [69,70,71]. Ricin A, the subunit with RNA N-glycosidase catalytic activity for 28S rRNA, was chosen as a cargo in this study since it rapidly shuts off protein synthesis thus preventing HIV-1 protein translation and leading to subsequent cell death [38]. The polymer shell of ricin A nanocapsules shields the cells from non-specific cytotoxicity before release and protects the cell similar to the non-encapsulated ricin A chain which is deficient for entering cells but retains catalytic activity. Furthermore, the polymer shell can shield its cargo from the environment [59,60,72], and thus, likely limits immune response and hepatic toxicity.

The most widely studied approach to purge reservoirs is to activate HIV-1 from latently infected cells during ART causing the death of the cells through HIV-1 induced death, natural cell progression, or immune responses. However, ART may not fully suppress HIV-1 before the cell dies. In this case, new HIV-1 will be produced and spread, thus replenishing or even increasing the latent reservoir. This novel HIV-1 PR- senstive nanocapsule can target the cell harboring newly activated HIV-1 and rapidly shut off protein synthesis, followed by killing of the cells, therefore limiting the time for HIV-1 replication after reactivation. Since the nanocapsule release is dependent upon sufficient protease expressed in the cell, it is likely that some infectious virus will be produced prior to inhibition of viral protein synthesis; however, our data show that much less virus would be released than is typically the case after latency reactivation. For clinical practice, the use of anti-retroviral agents would block the spread of any released HIV-1 prior to shutdown of HIV-1 translation. While ricin A was chosen as our focus here, any agent which either kills cells or interferes with the late stages of the HIV-1 life cycle could be substituted as a cargo within the nanocapsule. For example, DNA cassettes directed to HIV-1 RNA could be used in place of the luciferase DNA cassette shown here.

Additional in vivo studies will be required to ensure therapeutically effective and safe use. Several means are designed to ensure safety: use of the non-cell penetrating ricin A chain, shielding by the polymer shell, and HIV-1 protease dependent release. In culture, both in cell lines and primary T-cells, no obvious off target toxicities are evident, however, further studies in animals will provide a more stringent test of any potential off target toxicities. Further, the nanocapsule shell is likely to be not immunogenic [73], however the peptide crosslinkers may be immunogenic and will need to be assessed in vivo. We envision that future use of this strategy would be in combination with latency reactivating agents where this nanocapsule will release toxin in cells reactivated to produce HIV-1 throughout the body, killing those cells and thereby purging HIV-1 reservoirs.

## Supporting Information

S1 Appendix Materials and Methods. (DOCX)

S1 Fig Extracellular p24 concentration in J-Lat culture supernatant after prostratin reactivation. (DOCX)

S2 Fig The transduction efficiency of n-ricinA in J-Lat and control Jurkat cells, respectively. (DOCX)

S3 Fig Elimination of U1 cells with reactivation of HIV-1 with prostratin treatment. (DOCX)

S1 Table Amino acid sequences of peptide crosslinkers. (DOCX)

S2 Table P24 level in culture supernatant of U1 cell on Day 2. (DOCX)
