# Supplementary material for: Specific Elimination of Latently HIV-1 Infected Cells Using HIV-1 Protease-Sensitive Toxin Nanocapsules
Source: PLoS One. 2016 Apr 6;11(4):e0151572. doi: 10.1371/journal.pone.0151572 (PMC4822841; doi:10.1371/journal.pone.0151572)
Supplement: S1 Appendix — (DOCX) [file pone.0151572.s001.docx]

**SUPPORTING INFORMATION**

**Specific elimination of latently HIV-1 infected cells using HIV-1 protease-sensitive toxin nanocapsules**

Jing Wen^1^, Ming Yan^1,#^, Yang Liu^3^, Jie Li^3^, Yiming Xie^1^, Yunfeng Lu^3,4^, Masakazu Kamata^2,*^, Irvin S.Y. Chen^1,5,*^

**1** Department of Microbiology, Immunology, and Molecular Genetics,

**2** Division of Hematology-Oncology, David Geffen School of Medicine at University of California, Los

Angeles, Los Angeles, California, United States of America.

**3** Department of Biomolecular and Chemical Engineering, University of California Los Angeles,

**4** California NanoSystems Institute (CNSI), University of California Los Angeles,

**5** UCLA AIDS Institute, Los Angeles, California, United States of America.

# Present address: Calimmune, Inc., Pasadena, California, United States of America

**Correspondences**

*Masakazu Kamata, Department of Hematology-Oncology, University of California David Geffen School of Medicine, 615 Charles E. Young Dr. South, BSRB 157(11), Los Angeles, CA 90095, USA; e-mail: masa3k@ucla.edu

*Irvin SY Chen, Department of Microbiology, Immunology and Molecular Genetics, and Medicine, David Geffen School of Medicine at UCLA, 615 Charles E. Young Dr. South, BSRB 173, Los Angeles, CA 90095, USA; e-mail: syuchen@mednet.ucla.edu

**MATERIALS AND METHODS**

Materials

Reagents and solvents for synthesis of nanocapsules were purchased from Sigma-Aldrich (St. Louis, MO) and used as received without further purification unless otherwise noted. Roswell Park Memorial Institute (RPMI) 1640 Medium and Penicillin/Streptomycin/Glutamine were purchased from Invitrogen (Carlsbad, CA). Fetal Bovine Serum (FBS) was obtained from Omega Scientific Inc (Tarzana, CA). Fluorescence dye Rhodamine B isothiocyanate was purchased from Sigma-Aldrich (St. Louis, MO). Ricin A protein, prostratin and Saquinavir were purchased from Sigma-Aldrich (St. Louis, MO). Recombinant HIV-1 PR was obtained from MyBioSource. Purified HIV-2 protease protein and HIV-1 PR inhibitor Indinavir Sulfate (IDV) were provided by the NIH AIDS Reagent Program. HIV-1 PR FRET substrate was ordered from ANASPEC Peptide (Fremont, CA). The HIV-1 PR cleavable peptide and MMP cleavable peptide were purchased form Biomatik (Wilmington, DE).

Instruments

Dynamic light scattering (DLS) studies of the nanocapsules was measured on Zetasizer Nano instrument (Malvern Instruments Ltd, United Kingdom) equipped with a 10 mW helium-neon laser and thermoelectric temperature controller. Transmission electron microscope (TEM) images were obtained on a Philips CM120 electron microscope operating with an acceleration voltage of 120 kV. Luminescence intensities were measured with a FLUOstar Optima microplate reader, while fluorescence intensities were measured by VersaMax™ ELISA Microplate Reader. The cell count test was operated on MACSQuant flow cytometer (Miltenyi Biotec Inc.). The specific killing of nanocapsules was confirmed on BD LSRFortessa flow cytometer (BD Biosciences). Protein electrophoresis was run on Bio-Rad electrophoresis Chambers (Bio-Rad Laboratories, Inc.).

Cell cytotoxicity assay

Dead cells of J-Lat were first stained and removed by MACS^®^ LS columns. Cell cytotoxicity was tested by CytoTox-Glo^TM^ cytotoxicity kit from Promega. Briefly speaking, cells were seeded in 24-well plates at a density of 10^5^ cells per well, and transduced with nanocapsules and native protein ricin A for 4 hours. Then medium was removed and CytoTox-Glo substrate AAF-Glo was added to the wells. Luminescence from dead cells was measured by FLUOstar Optima microplate reader. The lysis reagent was added and luminescence from total cells was measured. The cytotoxicity was calculated as Dead cell luminescence/ Total cell luminescence X 100%.

*In vitro* test for specific release from nanocapsules

Nanocapsules were suspended in HIV-1 PR working buffer, which is 50 mM sodium acetate buffer containing 1 mM EDTA, 2.5 mM dithiothreitol (DTT), 1 M NaCl, 2.5% glycerol, 5% DMSO, and 0.1% NP-40, and treated with 250 ng (50uM) HIV PR at 37°C for 2 hours. After treatment, samples were denatured at 95°C with DTT, and applied to SDS-PAGE for protein electrophoresis.

HIV-1-infected 293-Affinofile with luciferase reporter gene

HIV-1-infected 293-Affinofile cells were seeded into 96-well plates at a density of 10^4^ cells per well with or without 1 ug/mL HIV-1 PR inhibitor (Saquinavir) for 2 hours. Then DNA cassette nanocapsules were added and incubated with the cells for 4 hours. After 2 days, the cells were lysed and luciferase activity was assayed with Dual-Glo Luciferase assay (Promega) using FLUOstar Optima microplate reader. The DNA cassette against luciferase was constructed under the human H1 RNA polymerase III promoter ([1](#_ENREF_3)).

FRET assay

One million J-Lat cells at different time points after reactivation were suspended in 100 µL of 200 mM HIV-1 PR working buffer and sonicated to extract cytoplasmic fractions. Following a 6000-rpm centrifugation for 5 min, 50 µL of the supernatant was mixed with 0.05 mM FRET-based fluorogenic substrate, and incubated at 37°C for 2 hours. The fluorescent intensity from the substrate was monitored at 490 nm by VersaMax™ ELISA Microplate Reader.

P24 antigen test

To confirm the virus production after reactivation, one million J-Lat cells were seeded in 24-well plates and reactivated with 10 µM prostratin. Culture supernatants were collected at different time points and filtrated with a 0.45 µm filter. To measure the effect of nanocapsules on P24 production, cells were first transduced with nanocapsules, native protein, or immunotoxin for 4 hours, and then stimulated with 10 µM prostratin. After one day, culture supernatant was collected and filtrated with a 0.45 µm filter. U1 cells were seeded in 24-well plates at a density of 10^6^ cells per well. Two days after reactivation with 10 µM prostratin, culture supernatant was collected and filtrated with a 0.45 µm filter. Culture supernatant was diluted in TritonX to a final concentration of 0.5%. Amount of p24 antigen was quantitatively measured by ELISA.

References

1. Liang M, Kamata M, Chen KN, Pariente N, An DS, Chen IS. Inhibition of HIV-1 infection by a unique short hairpin RNA to chemokine receptor 5 delivered into macrophages through hematopoietic progenitor cell transduction. J Gene Med. 2010;12(3):255-265.

2. Vandergeeten C, Quivy V, Moutschen M, Lint CV, Piette J, et al. HIV-1 protease inhibitors do not interfere with provirus transcription and host cell apoptosis induced by combined treatment TNF-α + TSA. Biochem Pharmacol. 2007; 73:1738-1748.

3. Lan J, Yang K, Byrd D, Hu N, Amet T, et al. Provirus Activation Plus CD59 Blockage Triggers Antibody-Dependent Complement-Mediated Lysis of Latently HIV-1−Infected Cells. J Immunol. 2014; 193:3577-3589.

4. Al-Harthi L, Roebuck KA, Kessler H, Landay A. Inhibition of Cytokine-Driven Human Immunodeficiency Virus Type 1 Replication by Protease Inhibitor. J Infect Dis. 1997; 176: 1175-1179.
