# Supplementary material for: Specific Elimination of Latently HIV-1 Infected Cells Using HIV-1 Protease-Sensitive Toxin Nanocapsules
Source: PLoS One. 2016 Apr 6;11(4):e0151572. doi: 10.1371/journal.pone.0151572 (PMC4822841; doi:10.1371/journal.pone.0151572)
Supplement: S1 Fig — (DOCX) [file pone.0151572.s002.docx]

**
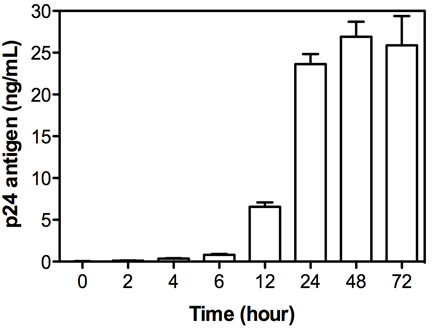
**

Figure S1. Extracellular p24 antigen concentration in J-Lat culture supernatant after prostratin reactivation. One million J-Lat cells were cultured in 500 µL medium with 10 µM of prostratin, and levels of p24 antigen in the culture supernatant were measured by ELISA.
