# Supplementary material for: Specific Elimination of Latently HIV-1 Infected Cells Using HIV-1 Protease-Sensitive Toxin Nanocapsules
Source: PLoS One. 2016 Apr 6;11(4):e0151572. doi: 10.1371/journal.pone.0151572 (PMC4822841; doi:10.1371/journal.pone.0151572)
Supplement: S2 Fig — (DOCX) [file pone.0151572.s003.docx]

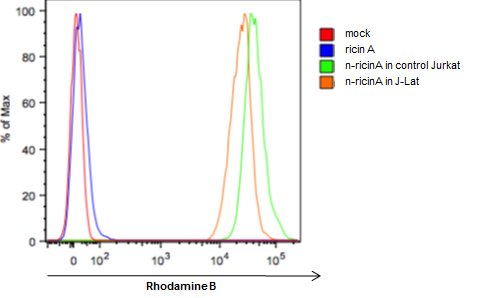


Figure S2. The transduction efficiency of n-ricinA in J-Lat and control Jurkat cells, respectively. Fluorescently labeled nanocapsules with Rhodamine B were separately incubated with either J-Lat or control Jurkat cells for 4 hours. The transduction efficiency was estimated to be 100% by the fluorescent intensity increase.
