# Supplementary material for: Specific Elimination of Latently HIV-1 Infected Cells Using HIV-1 Protease-Sensitive Toxin Nanocapsules
Source: PLoS One. 2016 Apr 6;11(4):e0151572. doi: 10.1371/journal.pone.0151572 (PMC4822841; doi:10.1371/journal.pone.0151572)
Supplement: S3 Fig — (DOCX) [file pone.0151572.s004.docx]

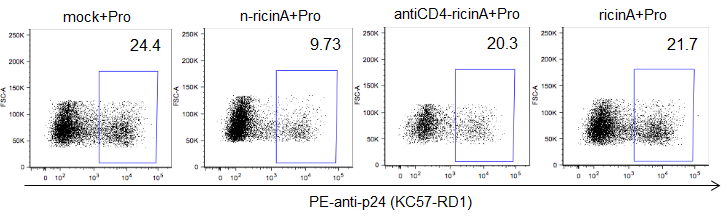


Figure S3. Elimination of U1 cells with reactivation of HIV-1 with prostratin treatment. One day after 10 µM prostratin reactivation, intracellular p24 antigen was stained by anti-p24 antibody and analyzed by flow cytometry.
