# Supplementary material for: Specific Elimination of Latently HIV-1 Infected Cells Using HIV-1 Protease-Sensitive Toxin Nanocapsules
Source: PLoS One. 2016 Apr 6;11(4):e0151572. doi: 10.1371/journal.pone.0151572 (PMC4822841; doi:10.1371/journal.pone.0151572)
Supplement: S1 Table — (DOCX) [file pone.0151572.s005.docx]

**Table S1** Amino acid sequences of peptide crosslinkers

| Sensitive to | Amino acid sequences |
| --- | --- |
| HIV-1 Protease (HIV-1 PR) | KVSQNYPIVK |
| Matrix metallopeptidase (MMP) | KVPLGVRTK |
