# Supplementary material for: Specific Elimination of Latently HIV-1 Infected Cells Using HIV-1 Protease-Sensitive Toxin Nanocapsules
Source: PLoS One. 2016 Apr 6;11(4):e0151572. doi: 10.1371/journal.pone.0151572 (PMC4822841; doi:10.1371/journal.pone.0151572)
Supplement: S2 Table — (DOCX) [file pone.0151572.s006.docx]

**Table S2** p24 level in culture supernatant of U1 cell on Day 2

| Transduced with | mock | n-ricinA | n-ricinA+IDV | antiCD4-ricinA | ricinA |
| --- | --- | --- | --- | --- | --- |
| p24 antigen (ng/mL) | 20.02 | 4.23 | 0.77* | 1.62 | 16.30 |

* With the HIV-1 protease inhibitor IDV, the p24 level of reactivated U1 cells was decreased, because IDV efficicently inhibited the release of mature virus(2-4).
